# Effect of Targeted Messaging on Return to In-Person Visits During the COVID-19 Pandemic: A Randomized Clinical Trial

**DOI:** 10.1001/jamanetworkopen.2021.15211

**Published:** 2021-06-30

**Authors:** Anne R. Cappola, Emily R. Schriver, Danielle L. Mowery, Colin Wollack, Camelot T. Ives, Ryan Gonzales, Joseph N. Cappella

**Affiliations:** 1 Division of Endocrinology, Diabetes, and Metabolism, University of Pennsylvania Perelman School of Medicine, Philadelphia; 2 Data Analytics Center, University of Pennsylvania Health System, Philadelphia; 3 Institute for Biomedical Informatics, University of Pennsylvania Perelman School of Medicine, Philadelphia; 4 Information Services, University of Pennsylvania Health System, Philadelphia; 5 Marketing, University of Pennsylvania Health System, Philadelphia; 6 Annenberg School for Communication, University of Pennsylvania, Philadelphia

## Abstract

This randomized clinical trial assess whether targeted messaging could improve the return to in-person visits during the COVID-19 pandemic.

## Introduction

During the COVID-19 pandemic, many patients have delayed care.^1,2,3,4^ We performed a randomized clinical trial to determine whether targeted messaging could improve the return to in-person visits.

## Methods

A randomized, double-blind, clinical trial (1:9 no letter vs letter; 1:1 tailored vs standard letter, 1:1 electronic vs mail) was conducted at 3 hospitals in the University of Pennsylvania Health System (eFigure and eAppendix in the Supplement 1). The University of Pennsylvania institutional review board deemed the study exempt and waived the need for consent because they found minimal risk to patient privacy. This study followed the Consolidated Standards of Reporting Trials (CONSORT) reporting guideline.

We identified adult patients aged 18 years or older who had canceled in-person appointments, procedures, or surgical procedures from March 9 through June 7, 2020, and had not rescheduled with clinicians in the Heart and Vascular Service Line and Penn Orthopaedics. Patients were randomized to groups that received no letter, a tailored letter, or a standard letter inviting them to reschedule canceled appointments. The tailored letter used an inoculation strategy in which patients’ perceived threats to safety were acknowledged and addressed early in the message,^5,6^ with age specificity for patients 65 years or older and informational and word count equivalence between letters. The protocol and letter copies are included in Supplement 2. Messages were sent to patients on June 12 through June 17, 2020, with a final follow-up on July 17, 2020.

Patients self-reported their race based on the following predefined categories: White, Black, Asian, Pacific Islander, American Indian, or other. Only data from White patients and Black patients were included because of the small number of patients who identified with the other categories listed for race. Race was included in the study because prior data suggested that there were differences in access to health care between Black individuals and White individuals during the COVID-19 pandemic.

The primary outcome was the percentage of patients who had an in-person visit, procedure, or surgery with a clinician in the service line within 1 month of sending the letter. Secondary outcomes included the percentage of patients who had a telemedicine visit within 1 month or who scheduled a visit (in-person or telemedicine) within 1 month for any future date.

We estimated a 90% power to detect a 2% difference between letter types and a 4% difference between delivery methods. The control group was compared with all groups combined, with letter types, and with delivery methods for each outcome. A χ^2^ analysis was performed, with pairwise comparisons adjusted by the Holm method. The percentage of patients who scheduled a future visit were compared by letter type and by delivery type overall and stratified by age, sex, race, median household income, insurance type, and service line, with testing for interactions. Analyses were conducted using R, version 3.63 (R Project for Statistical Computing). *P* values were 2-sided, and statistical significance set at *P* < .05.

## Results

Of 38 493 patients with cancellations, 27 373 rescheduled and 11 120 (28.9%) were randomized (Figure). The median age was 59.7 (range, 18-100) years, 6074 (55%) were women, and 6830 (61%) were White patients.

**Figure.**
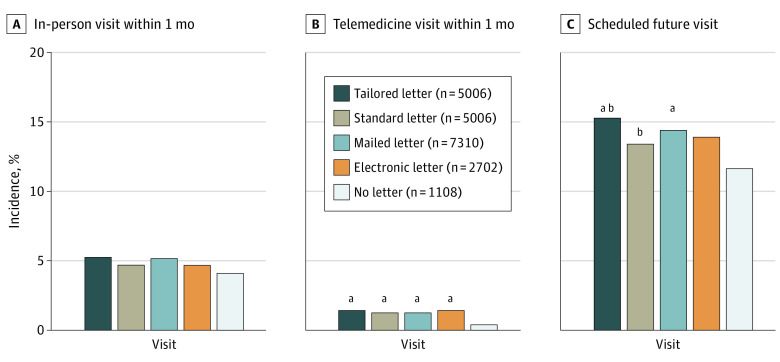
Incidence of Outcomes by Communication Type For each outcome, the control group (no letter) was compared with all of the intervention groups combined, with tailored and standard letters groups, and with mailed and electronic delivery methods. Comparisons by χ^2^ analysis were performed first. When *P* was <.05 by χ^2^ analysis, pairwise comparisons were performed. ^a^*P* < .05 for pairwise comparison with the no letter group, adjusted via the Holm method. ^b^*P* < .05 for pairwise comparison within the letter group, adjusted via the Holm method.

In-person return visits within 1 month were low and not significantly different between patients who did and did not receive a letter (503 [5.0%] vs 45 [4.1%]; *P* = .18) (Figure). Patients receiving any letter vs no letter had a higher percentage of telemedicine visits and future scheduled visits within 1 month (telemedicine visits: 132 [1.3%] vs 4 [0.4%]; *P* = .006; future visits: 1439 [14.4%] vs 130 [11.7%], *P* = .02).

Patients receiving the tailored letter vs standard letter scheduled more visits, achieving statistical significance overall (768 [15.3%] vs 671 [13.4%]; *P* = .006) and for individuals younger than 65 years, women, Black patients, White patients, patients with commercial insurance, patients with lower household income, and patients receiving orthopedic services (Table). For the subset randomized by delivery type, patients with a household income below the median were more likely to schedule a visit when mailed a letter vs receiving an electronic letter, with a statistically significant interaction by household income (230 [16.6%] vs 177 [13.0%]).

**Table.** Incidence of Scheduled Visits by Communication Type and Patient Characteristics

Characteristic	No./total No. (%)		*P* value^a^	No./total No. (%)		*P* value^a^
	Tailored letter	Standard letter		Mailed letter	Electronic letter	
Overall	768/5006 (15.3)	671/5006 (13.4)	.006	421/2702 (15.6)	378/2702 (14.0)	.10
Age, y						
<65	387/2829 (13.7)	311/2829 (11.0)	.002	221/1594 (13.9)	198/1594 (12.4)	.23
≥65	381/2177 (17.5)	360/2177 (16.5)	.40	200/1108 (18.1)	180/1108 (16.2)	.26
Sex						
Men	337/2256 (14.9)	304/2291 (13.3)	.11	187/1184 (15.8)	181/1189 (15.2)	.70
Women	431/2750 (15.7)	367/2715 (13.5)	.02	234/1518 (15.4)	197/1513 (13.0)	.06
Race						
White	467/3029 (15.4)	420/3123 (13.4)	.03	301/1935 (15.6)	266/1918 (13.9)	.14
Black	233/1360 (17.1)	185/1334 (13.9)	.02	86/496 (17.3)	79/532 (14.8)	.28
Insurance status						
Commercial	329/2400 (13.7)	264/2394 (11.0)	.005	215/1546 (13.9)	182/1489 (12.2)	.17
Medicare or Medicaid	427/2535 (16.8)	401/2542 (15.8)	.30	205/1146 (17.9)	192/1189 (16.1)	.26
Household income^b^^,^^c^						
Below median	398/2541 (15.7)	342/2530 (13.5)	.03	191/1320 (14.5)	201/1342 (15.0)	.71
At or above median	370/2465 (15.0)	329/2476 (13.3)	.08	230/1382 (16.6)	177/1360 (13.0)	.008
Clinical service^d^						
Cardiac	440/2799 (15.7)	431/2799 (15.4)	.74	252/1492 (16.9)	230/1492 (15.4)	.27
Orthopedic	328/2207 (14.9)	240/2207 (10.9)	<.001	169/1210 (14.0)	148/1210 (12.2)	.21

^a^ *P* values for comparisons by χ^2^ analysis.

^b^ The median household income was determined from the patient’s zip code and was $72 157 overall and $77 695 for the subset randomized to mailed or electronic letter.

^c^ *P* for interaction = .03 between mailed and electronic letters.

^d^ *P* for interaction = .004 between tailored and standard letters.

## Discussion

In this randomized clinical trial, we found that a large proportion of patients who canceled visits and procedures early in the COVID-19 pandemic did not reschedule once reopening occurred. A single message targeted directly to these patients did not affect the return to in-person visits within 1 month, but it resulted in a small increase in reengagement through telemedicine and rescheduling of future visits. Study limitations include limited outcomes and unknown generalizability to other locations and times. Strategies to improve patient engagement, with attention to message framing and delivery mode, are needed to encourage continuity of health care in the era of COVID-19.

## Notes

Supplement 1. eMethods. **eFigure.** Study Design

Supplement 2. Protocol and Letter Copies

Supplement 3. Data Sharing Statement
