# Supplementary material for: Effect of Targeted Messaging on Return to In-Person Visits During the COVID-19 Pandemic: A Randomized Clinical Trial
Source: JAMA Netw Open. 2021 Jun 30;4(6):e2115211. doi: 10.1001/jamanetworkopen.2021.15211 (PMC8246312; doi:10.1001/jamanetworkopen.2021.15211)
Supplement: Supplement 1. — eMethods. eFigure. Study Design [file jamanetwopen-e2115211-s001.pdf]

Supplemental Online Content

Cappola AR, Schriver ER, Mowery DL, et al. Effect of targeted messaging on return to in-person visits during the COVID-19 pandemic: a randomized clinical trial. *JAMA Netw Open*. 2021;4(6):e2115211. doi:10.1001/jamanetworkopen.2021.15211

eMethods.

eFigure. Study Design

This supplemental material has been provided by the authors to give readers additional information about their work.

© 2021 Cappola AR et al. *JAMA Network Open.*

eMethods.

The first COVID patient was announced in Philadelphia on March 10, 2020. A stay-at-home order was instituted on March 23, 2020 and changed to a safer-at-home advisory on June 5, 2020. Hospitalization rates and deaths peaked in Philadelphia during the week of April 12, 2020. Patients who had canceled in-person appointments, procedures, or surgeries from March 9 through June 7, 2020, and not rescheduled with clinicians in the Heart and Vascular Service Line and Penn Orthopaedics were identified. Patients who had accessed the electronic health record messaging portal within 6 months were randomized into a control group receiving no intervention or to receive either a tailored or a standard letter inviting rescheduling, delivered via either the messaging portal or mail (eFigure). Patients who had not enrolled in or had not accessed the portal within 6 months were randomized to the control group or to receive a tailored or standard letter delivered via mail.

Race was self-reported based on the following predefined categories: White, Black, Asian, Pacific Islander, American Indian, or Other. Only White or Black patients were included in the Table due to small numbers in the other categories. Race was included in the study due to prior data suggesting differences between Blacks and Whites in accessing care during the pandemic.

© 2021 Cappola AR et al. *JAMA Network Open.*

eFigure. Study Design

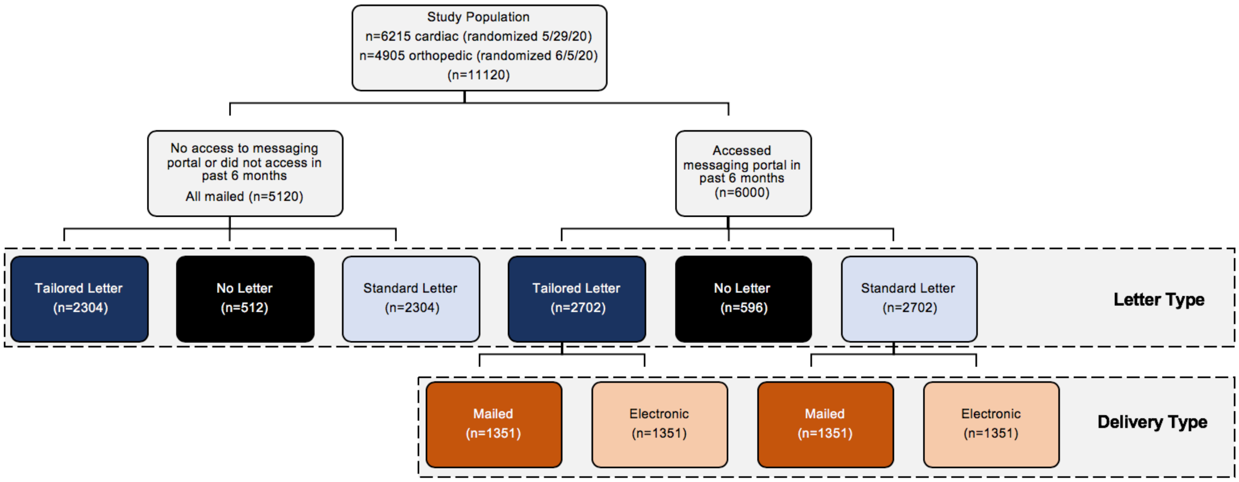

The study population was initially stratified based on access to the messaging portal of the electronic health record. All patients were randomized by letter type to no letter (10%), a tailored letter (45%), or a standard letter (45%). Patients who had accessed the messaging portal within the past 6 months were additionally randomized 1:1 by delivery type, electronically or by mail. Patients who had not accessed the messaging portal within the past 6 months or had not enrolled in the messaging portal received the letter by mail.

© 2021 Cappola AR et al. *JAMA Network Open.*
