# Supplementary material for: Effect of Targeted Messaging on Return to In-Person Visits During the COVID-19 Pandemic: A Randomized Clinical Trial
Source: JAMA Netw Open. 2021 Jun 30;4(6):e2115211. doi: 10.1001/jamanetworkopen.2021.15211 (PMC8246312; doi:10.1001/jamanetworkopen.2021.15211)
Supplement: Supplement 2. — Protocol and Letter Copies [file jamanetwopen-e2115211-s002.pdf]

Comparison of the Effectiveness of Communication Strategies on the Return to In-Person Visits to a Medical Center After the Emergence of COVID-19: A Randomized Trial 5/30/20

PI's
Anne R. Cappola, M.D., Sc.M.
Division of Endocrinology, Diabetes, and Metabolism
Smilow TRC 12, Room 136
Perelman School of Medicine
215-573-5359
acappola@pennmedicine.upenn.edu

Joseph N. Cappella, PhD
Gerald R. Miller Professor of Communication
Annenberg School for Communication
3620 Walnut St.
University of Pennsylvania
215-898-7059
Joseph.cappella@asc.upenn.edu

1. Background and study rationale

In mid-March 2020, the University of Pennsylvania Hospital System (UPHS) instituted a series of initiatives to expand in-patient capacity for a surge of COVID-19 patients and to reduce the risk of transmission of COVID-19 from in-person visits. These efforts resulted in a 90% reduction in surgical procedures and 80% reduction in outpatient visits to UPHS sites. With the stabilization of COVID-19 cases within UPHS, plans are underway to resume surgical procedures and in-person visits. However, a recent national survey showed that only 15% of patients would be comfortable entering a local hospital for a medical procedure immediately after COVID-19 restrictions were lifted. Major concerns included risk of getting COVID-19 from other patients, lack of appropriate cleaning procedures, lack of protective equipment, and medical costs. For many patients, a delay in diagnosis, treatment or preventive care will result in irreversible adverse health consequences that pose a greater risk to the patient than the risk of acquiring COVID-19 through a healthcare encounter. In addition, reluctance to return for in-person care may vary by level of education and race, magnifying existing health disparities.

We hypothesize that the use of established communication strategies shown in previous studies to be effective in behavioral change will increase the percentage of patients who present for in-person visits, compared with standard UPHS patient communications. We also hypothesize that mailed letters will be more effective than messages through the electronic health record (EHR), irrespective of the content.

2. Study objectives

2.1 Primary objectives

1. To compare two letters whose content has different approaches to patient communication on the percentage of contacted patients who have an in-person visit or procedure at a UPHS site.
2. To compare distribution of letter content via a message through the EHR to a mailed letter on the percentage of contacted patients who have an in-person visit or procedure at a UPHS site.

2.2 Secondary objectives

To compare each of these approaches to patient communication on the following secondary outcomes:

- Percentage of patients who schedule a visit
- Percentage of patients who complete a telemedicine visit

- Percentages of patients who have each outcome stratified by age, sex, race, ethnicity, income (estimated from zip code), type of insurance, and primary encounter service line

3. General design

Randomized, double-blind, factorial design of intervention letter vs. standard of care letter and EHR vs mail delivery mechanism

3.1 Intervention and control

Preparations are currently underway to inform UPHS patients that the hospitals and practices are open to patients who have previously cancelled or postponed medical visits and procedures due to the emergence of COVID-19. We expect this message to function as the control message in our trial. The intervention message will modify this control message using an “inoculation strategy” in which patients’ perceived threats to safety are acknowledged and addressed early in the message. In addition, it will be personalized based on whether the patient is less than 65 years of age or 65 years of age or older. Informational equivalence and word count will be maintained between control and intervention messages.

3.2 Randomization

10% no extra communication vs. 90% extra communication
Within the group who have extra communication:
1:1 Standard UPHS message vs. customized message
1:1 EHR vs. mail for patients with EHR message capability; mail only for those without EHR message capability
Randomization will occur at the level of the patient.

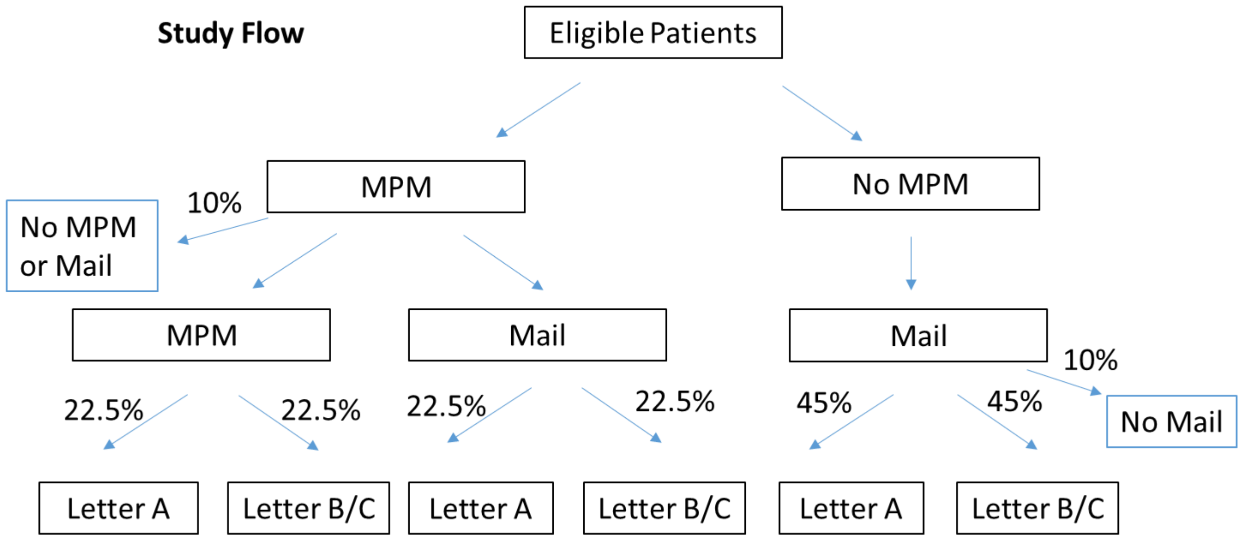

3.3 Primary study endpoint

The percentage of contacted patients who have an in-person visit or procedure at a UPHS site, including an inpatient or outpatient procedure or an in-person outpatient visit, within 1 month of contact.

3.4 Secondary study endpoints

The percentage of patients who schedule a visit within 1 month of contact.
The percentage of patients who complete a telemedicine visit within 1 month of contact.

4 Study population

4.1 Major inclusion criteria

Patients with a cancellation or failure to show for a procedure or visit from March 9, 2020 through June 7, 2020 (90 days) and who have not rescheduled as of the date of randomization
All patients were scheduled patients in one of two service lines: Heart & Vascular and Orthopaedics.

4.2 Major exclusion criteria

Death

5. Study procedures

All patient lists will be generated and transferred behind Penn's firewall. A list of service line providers will be generated for each service line and given to an analyst at the Penn Data Analytics Center (DAC). For each service line, a list of patients will be generated who had a visit scheduled to occur with a service line provider from March 9, 2020 through June 7, 2020 (90 days) that was missed (cancelled or no show) and not rescheduled as of the date of the search. A separate list will be generated for patients who have accessed myPennMedicine (MPM) within the past 6 months. By service line, 10% of all patients who have accessed MPM in the past 6 months will be excluded from receiving an extra communication. The remaining 90% will be randomized to MPM or mail. Those randomized to MPM will be randomized to sending letter A (standard letter) or letter B/C (customized letter). The customized letter delivered will differ between those aged below 65 (letter B) and age 65 years or above (letter C). For those age 65 years or above, the line "You are in an age group where you may have specific concerns about your safety in returning for a visit." will be included. A PennChart analyst will design the code to send the MPM letters (A, B, or C) in bulk from each service line for those randomized to MPM distribution, according to randomization status.

Two additional lists of randomized patients and addresses will be generated, by service line:

1. MPM users who were randomized to receive a mailed letter, who will be randomized to letter A or letter B/C.
2. Non-MPM users, who will be randomized to letter A or letter B/C after excluding 10% as a control group.

AE Litho, which is used by Penn Medicine for mailing letters for the marketing department, will receive each randomization list for patients receiving letters and will mail the letters (A, B, or C) according to service line and letter assignment. The letters will not contain the patient name, provider name, patient diagnosis, or any protected health information.

Outcome data and data for stratified analyses will be extracted from PennChart by a DAC analyst, deidentified, and compiled into an analytic dataset.

5. Statistical plan

Planned enrollment: We estimate that we will include a minimum of 3,000 patients from each service line, for a minimum sample size of 6,000.

Power calculation: We estimate that 20% of the control group will reschedule. We estimate that we will have 90% power to detect a 2% absolute difference between letter A and letter B/C and with 50% active MPM use, a 4% absolute difference between MPM and a mailed letter.

We will use a chi-squared test to compare outcomes between groups based on randomization (intention to treat). Additional analyses will be stratified by age (< or ≥ 65 years), sex, race, ethnicity, income (estimated from zip code), type of insurance (commercial, Medicaid, Medicare, or none), and service line.

6. Risks and benefits

All members of the research team have completed CITI human subjects research training. Confidentiality risk will be minimized by deidentifying information after a clinical informatics analyst at the Penn Data Analytics Center extracts the outcome data from PennChart.

7. Informed consent

We are requesting a waiver of informed consent since this is low risk and impossible to evaluate with informed consent.

Control letter for Heart and Vascular (Page 6)
Tailored letter for Heart and Vascular, under age 65 (Page 7)
Tailored letter for Heart and Vascular, age 65 or older (Page 8)
Control letter for Penn Orthopaedics (Page 9)
Tailored letter for Penn Orthopaedics, under age 65 (Page 10)
Tailored letter for Penn Orthopaedics, age 65 or older (Page 11)

Dear Valued Patient,

We understand that you may be concerned about how to safely access health care in this environment. It is important that you have timely and high quality heart care. We are continuing to provide care to our patients during the COVID-19 pandemic.

At Penn Heart and Vascular, we are offering virtual video appointments through smartphones or computers and in-person visits for our patients who need follow-up care. Your provider will help determine if a virtual appointment or in-person visit is right for you. We are still performing heart and vascular surgeries, procedures, and outpatient testing. *To schedule an appointment, call 800-789-7366 or contact your provider's office directly.*

Our highest priority is making sure you get the care you need, when you need it and in the safest way possible. Whether it's at the hospital, the clinic or your provider's office, we want you to feel comfortable knowing that we have put processes in place for the safety of our patients. We are following guidance from the U.S. Centers for Disease Control and Prevention and have taken extra precautions, including:

- Rigorously cleaning and disinfecting several times per day, in accordance with infection control guidelines.
- Requiring masks or face coverings for all staff, patients and visitors.
- Maximizing physical distancing in waiting rooms and patient care areas.
- Initiating new processes such as checking-in and checking-out without having to come into physical contact with staff.

We have established a visitation policy which limits visitors as part of our safety protocols. However, visitors are allowed in certain circumstances that may apply to you. Please access visitor information here: https://www.pennmedicine.org/coronavirus/visitation-policy-and-guidelines

To limit COVID-19 exposure, valet service is currently closed at all Penn Medicine locations. Garage and outdoor parking, drop-offs, shuttle service and patient escorts are still available. Please access parking information here: https://www.pennmedicine.org/for-patients-and-visitors/penn-medicine-locations/parking

We want to assure you that our highest priority is making sure you get the care you need, when you need it, and in the safest way possible. Please don't hesitate to contact your provider with any questions or to schedule an appointment.

Sincerely,
Penn Medicine Heart & Vascular Center

Dear Valued Patient,

We noticed that you missed a recent appointment with your cardiovascular provider. While we can't be sure why you missed your appointment, we understand this is a challenging time and you may have questions or concerns about getting healthcare right now.

Safety: Your safety has always been a high priority for us, now more than ever. You can be confident that our goal is to provide care in the safest way possible to protect your health, and that of the staff, visitors, and other patients. Here are some of our safety steps:

- Patients and staff (and visitors)
  - wear masks or face coverings at all times
  - are screened at entry for symptoms
  - have their temperature taken without removing masks
- Physical distancing is created
  - through reconfigured waiting rooms
  - staggered scheduling
  - controlled elevator access
  - check-in and check-out without physical contact with staff
- Areas that patients use are thoroughly cleaned and disinfected throughout the day.
- Visitors are limited and valet parking is closed.

For more information, see https://www.pennmedicine.org/coronavirus

You may have specific concerns about your safety in returning to a Penn hospital for a visit or procedure. We understand your concerns. It is time to think about your heart and your health. Delaying your heart care can have serious consequences. If you are thinking that it is better to wait, we urge you to contact your provider and talk through your questions and concerns.

At Penn Heart and Vascular, we are offering virtual video appointments through smartphones or computers and in-person visits for our patients who need care. Your provider will help determine if a virtual appointment or in-person visit is right for you. We are still performing heart and vascular surgeries, procedures, and outpatient testing. Please contact your provider for more information. *To schedule an appointment, call 800-789-7366 or contact your provider's office directly.*

We are continuing to provide care to all of our patients during the COVID-19 pandemic. Heart disease cannot wait. Be assured that we will provide care in the safest way possible to protect your health.

Sincerely,
Penn Medicine Heart & Vascular Center

Dear Valued Patient,

We noticed that you missed a recent appointment with your cardiovascular provider. While we can't be sure why you missed your appointment, we understand this is a challenging time and you may have questions or concerns about getting healthcare right now.

Safety: Your safety has always been a high priority for us, now more than ever. You can be confident that our goal is to provide care in the safest way possible to protect your health, and that of the staff, visitors, and other patients. Here are some of our safety steps:

- Patients and staff (and visitors)
  - wear masks or face coverings at all times
  - are screened at entry for symptoms
  - have their temperature taken without removing masks
- Physical distancing is created
  - through reconfigured waiting rooms
  - staggered scheduling
  - controlled elevator access
  - check-in and check-out without physical contact with staff
- Areas that patients use are thoroughly cleaned and disinfected throughout the day.
- Visitors are limited and valet parking is closed.

For more information, see https://www.pennmedicine.org/coronavirus

You are in an age group where you may have specific concerns about your safety in returning for a visit or procedure. We understand your concerns. It is time to think about your health. Delaying your heart care can have serious consequences. If you are thinking that it is better to wait, we urge you to contact your provider and talk through your questions and concerns.

At Penn Heart and Vascular, we are offering virtual video appointments through smartphones or computers and in-person visits for our patients who need care. Your provider will help determine if a virtual appointment or in-person visit is right for you. We are still performing heart and vascular surgeries, procedures, and outpatient testing. Please contact your provider for more information. *To schedule an appointment, call 800-789-7366 or contact your provider's office directly.*

We are continuing to provide care to all of our patients during the COVID-19 pandemic. Heart disease cannot wait. Be assured that we will provide care in the safest way possible to protect your health.

Sincerely,
Penn Medicine Heart & Vascular Center

Penn Medicine
Orthopaedics

Dear Patient,

We understand that you may be concerned about how to safely access health care in this environment. It is important that you have timely and high quality orthopaedic care. We are continuing to provide care to our patients during the COVID-19 pandemic.

At Penn Orthopaedics, we are offering virtual video appointments through smartphones or computers and in-person visits for our patients who need follow-up care. Your provider will help determine if a virtual appointment or in-person visit is right for you. We are still performing surgeries, procedures, and outpatient testing.

To schedule an appointment, call 800-789-7366 or contact your provider's office directly.

Our highest priority is making sure you get the care you need, when you need it and in the safest way possible. Whether it is at the hospital or provider's office, we want you to feel comfortable knowing that we have put processes in place for the safety of our patients. We are following guidance from the U.S. Centers for Disease Control and Prevention and have taken extra precautions, including:

- Rigorously cleaning and disinfecting several times per day, in accordance with infection control guidelines.
- Requiring masks or face coverings for all staff, patients and visitors.
- Maximizing physical distancing in waiting rooms and patient care areas.
- Initiating new processes such as checking-in and checking-out without having to come into physical contact with staff.

We have established a visitation policy which limits visitors as part of our safety protocols. However, visitors are allowed in certain circumstances that may apply to you. Please access visitor information at PennMedicine.org: www.pennmedicine.org/coronavirus

To limit COVID-19 exposure, valet service is currently closed at all Penn Medicine locations. Garage and outdoor parking, drop-offs, shuttle service and patient escorts are still available. Please access parking information here: www.pennmedicine.org/coronavirus

We want to assure you that our highest priority is making sure you get the care you need, when you need it, and in the safest way possible. Please don't hesitate to contact your provider with any questions or to schedule an appointment.

Sincerely,
Penn Orthopedics

Penn Medicine
Orthopaedics

Dear Patient,

We noticed that you missed a recent appointment with your orthopaedic provider. While we can't be sure why you missed your appointment, we understand this is a challenging time and you may have questions or concerns about getting healthcare right now.

Safety: Your safety has always been a high priority for us, now more than ever. You can be confident that our goal is to provide care in the safest way possible to protect your health, and that of the staff, visitors, and other patients. Here are some of our safety steps:

- Patients and staff (and visitors)
  - wear masks or face coverings at all times
  - are screened at entry for symptoms
  - have their temperature without removing masks
- Physical distancing is created
  - through reconfigured waiting rooms
  - staggered scheduling
  - controlled elevator access
  - check-in and check-out without physical contact with staff
- Areas that patients use are thoroughly cleaned and disinfected throughout the day.
- Visitors are limited and valet parking is closed.

For more information, see www.pennmedicine.org/coronavirus

You may have specific concerns about your safety in returning to Penn Orthopaedics for a visit or procedure. We understand your concerns. It is time to think about taking care of yourself. Delaying your care can have unexpected consequences. If you are thinking that it is better to wait, we urge you to contact your provider and talk through your questions and concerns.

At Penn Orthopaedics, we are offering virtual video appointments through smartphones or computers and in-person visits for our patients who need care. Your provider will help determine if a virtual appointment or in-person visit is right for you. We are still performing surgeries, procedures, and outpatient testing. Please contact your provider for more information.

To schedule an appointment, call 800-789-7366 or contact your provider's office directly.

We are continuing to provide care to all of our patients during the COVID-19 pandemic. Your health should not wait. Be assured that we will provide care in the safest way possible to protect your health.

Sincerely,
Penn Orthopaedics

Penn Medicine
Orthopaedics

Dear Patient,

We noticed that you missed a recent appointment with your orthopaedic provider. While we can't be sure why you missed your appointment, we understand this is a challenging time and you may have questions or concerns about getting healthcare right now.

Safety: Your safety has always been a high priority for us, now more than ever. You can be confident that our goal is to provide care in the safest way possible to protect your health, and that of the staff, visitors, and other patients. Here are some of our safety steps:

- Patients and staff (and visitors)
  - wear masks or face coverings at all times
  - are screened at entry for symptoms
  - have their temperature without removing masks
- Physical distancing is created
  - through reconfigured waiting rooms
  - staggered scheduling
  - controlled elevator access
  - check-in and check-out without physical contact with staff
- Areas that patients use are thoroughly cleaned and disinfected throughout the day.
- Visitors are limited and valet parking is closed.

For more information, see www.pennmedicine.org/coronavirus

You are in an age group where you may have specific concerns about your safety in returning for a visit or procedure. We understand your concerns. It is time to think about your health. Delaying your care can have consequences. If you are thinking that it is better to wait, we urge you to contact your provider and talk through your questions and concerns.

At Penn Orthopaedics, we are offering virtual video appointments through smartphones or computers and in-person visits for our patients who need care. Your provider will help determine if a virtual appointment or in-person visit is right for you. We are still performing surgeries, procedures, and outpatient testing. Please contact your provider for more information.

To schedule an appointment, call 800-789-7366 or contact your provider's office directly.

We are continuing to provide care to all of our patients during the COVID-19 pandemic. Your health should not wait. Be assured that we will provide care in the safest way possible to protect your health.

Sincerely,
Penn Orthopaedics

162
