# Supplementary material for: Effect of Targeted Messaging on Return to In-Person Visits During the COVID-19 Pandemic: A Randomized Clinical Trial
Source: JAMA Netw Open. 2021 Jun 30;4(6):e2115211. doi: 10.1001/jamanetworkopen.2021.15211 (PMC8246312; doi:10.1001/jamanetworkopen.2021.15211)
Supplement: Supplement 3. — Data Sharing Statement [file jamanetwopen-e2115211-s003.pdf]

Data Sharing Statement

Cappola. Effect of Targeted Messaging on Return to In-Person Visits During the COVID-19 Pandemic. *JAMA Netw Open*. Published June 30, 2021. doi:10.1001/jamanetworkopen.2021.15211

Data
Data available: No
